# The role of reporting standards for metabolite annotation and identification in metabolomic studies

**DOI:** 10.1186/2047-217X-2-13

**Published:** 2013-10-16

**Authors:** Reza M Salek, Christoph Steinbeck, Mark R Viant, Royston Goodacre, Warwick B Dunn

**Affiliations:** 1 European Molecular Biology Laboratory-European Bioinformatics Institute (EMBL-EBI), Wellcome Trust Genome Campus, Hinxton CB10 1, Cambridgeshire, SD, UK; 2 Department of Biochemistry and Cambridge Systems Biology Centre, University of Cambridge, Cambridge CB2 1, GA, UK; 3 School of Biosciences, The University of Birmingham, Edgbaston, Birmingham, B15 2TT, UK; 4 School of Chemistry, Manchester Institute of Biotechnology, University of Manchester, Manchester M1 7DN, UK

## Abstract

The application of reporting standards in metabolomics allow data from different laboratories to be shared, integrated and interpreted. Although minimum reporting standards related to metabolite identification were published in 2007, it is clear that significant efforts are required to ensure their continuous update and appropriate use by the metabolomics community. These include their use in metabolomics data submission (e.g., MetaboLights) and as a requirement for publication in peer-reviewed journals (e.g., *Metabolomics*). The Data Standards and Metabolite Identification Task Groups of the international Metabolomics Society are actively working to develop and promote these standards and educate the community on their use.

## Background

Metabolomics studies focus on the investigation of the complex and dynamic biochemical interactions of metabolites, both with other biochemicals and their environment [1]. Targeted and non-targeted studies are applied and each impacts differently on the task of metabolite identification [1]. Non-targeted studies are applied to study tens to thousands of different metabolites in a single sample without the chemical identification of metabolites known prior to the study; data acquired during or after the study are applied to annotate or to identify metabolites, and this is widely regarded as a significant bottleneck (see [2]). This bottleneck is not observed for targeted studies where the chemical identity is known prior to the study. It is vital that robust annotation or identification of metabolites in non-targeted studies is performed to maximise their interpretation and impact. With robust annotation or identification, biological interpretation of data can be performed for a single study and data from different studies performed in the same laboratory or different research groups can be compared. However, it is important that methods applied in annotation or identification are suitably described so that the confidence of each chemical annotation or identification can be quantified. In metabolomics, the importance of reporting standards, in other words minimal information checklists to ensure the reporting of the same core set of information, was recognised early and these were developed.

### Standards for reporting metabolite annotation and identification

The Metabolomics Standards Initiative (MSI) was conceived in 2005 following earlier work by the Standard Metabolic Reporting Structure initiative and the Architecture for Metabolomics consortium [3]. The early efforts of MSI were focused on community-agreed reporting standards, which provided a clear description of the biological system studied and all components of metabolomics studies. The aim was to allow data to be efficiently applied, shared and reused. There were five working groups and the chemical analysis group proposed minimum information for reporting chemical analysis, including minimum metadata to report related to metabolite identification [4].

The chemical analysis working group defined four different levels of metabolite identification observed in the scientific literature. These included identified metabolites (level 1), putatively annotated compounds (level 2), putatively characterised compound classes (level 3), and unknown compounds (level 4). There are important differences between these levels. Level 1 identification necessitates that 2 or more orthogonal properties of an authentic chemical standard analysed in the researcher’s laboratory are compared to experimental data acquired in the same laboratory with the same analytical methods. By contrast, level 2 and 3 annotation does not require matching to data for authentic chemical standards acquired within the same laboratory. Many studies do not compare experimental data to data acquired for authentic chemical standards, and therefore annotations and not identifications are achieved. Defining metabolites as identified or annotated is hugely important to provide clarity. It is recommended that all researchers define the level of identification, common name and structural code (e.g., InChI or SMILES) in their publications and when submitting data to repositories. However, the current use of these standards is low in peer-reviewed publications. Out of 20 randomly chosen metabolomics studies published in peer-reviewed journals in 2013, only six articles defined how metabolites were annotated or identified, only one article included relevant metadata, and no articles defined the level applied. As a community we need to robustly apply these reporting standards routinely.

### Current initiatives and the road ahead

Although community-agreed reporting standards were published in 2007 there is clearly still much to do to ensure these standards are applied by all metabolomics researchers. There are a number of key groups and initiatives that were recently established or are currently developing who will assist in enabling and ensuring these standards are further developed and applied. One such application of MSI guidelines is for reporting metabolites annotated or identified in data submitted to public metabolomic repositories. MetaboLights is the first general purpose database in metabolomics and became operational in 2012 [5]. MetaboLights adheres to MSI standards for metadata reporting and uses the ISA-tab format [6] to capture and study metadata, including the metabolites identified or annotated. Currently MetaboLights relies on authors for correct reporting of the metabolites identified on two levels. First, the correct intended chemical name is reported and mapped to an existing metabolite database (for example, ChEBI). Second, the level of confidence for correctly identifying a metabolite is defined; this is complex and technology platform dependent, presently MetaboLights follows earlier MSI guidelines and publications on metabolite identification reporting. Unknown compounds are tracked based on their analytical metadata, such as chemical shift for NMR and *m*/*z* for mass spectrometry.

COSMOS (Coordination of Standards in Metabolomics), was launched in October 2012, bringing together European data providers to set and promote community standards that will make it easier to disseminate metabolomics data through life science e-infrastructures [7]. COSMOS is working with, and builds on, existing initiatives such as the MSI, Metabolomics Society and the National Institutes of Health (NIH) Metabolomics Workbench [8] to update existing standards in metabolomics and to create missing standards, ensuring that community-accepted workflows for data exchange between repositories and laboratories are agreed. COSMOS will engage with publishers to agree on requirements for authors to deposit metabolomics results, as is required for other “omics” disciplines. For example, the Springer-published journal *Metabolomics*[9] is the official journal of the Metabolomics Society and for the last three years has encouraged authors to ensure their papers are as MSI compliant as possible [10]. It is appropriate for authors to be as transparent as possible in terms of reporting what was conducted in their studies, and this is good scientific practice. In the near future the journal will be testing approaches for assessing if papers are MSI-compliant in collaboration with the COSMOS project described above. The journal *Metabolomics* is also in discussion with database providers, including EBI MetaboLights and the NIH Metabolomics Workbench and in the future hopes that a transparent and user-friendly system will be applied where editors and referees can review metabolomics data and associated metadata during the paper reviewing process. Only through the application of these reporting standards in data repositories and the required deposition of study data and meta-data for publication in peer-reviewed journals will their use become routine.

As our appreciation of the complexity of metabolomics grows, the original MSI reporting standards require revisiting and possible modification to enhance the accuracy of reporting metabolite identification. The international Metabolomics Society has a key role to play to ensure data standards are further developed and applied effectively. The Data Standards and Metabolite Identification Task Groups were both initiated by the Metabolomics Society in 2013 to ensure standards are further evolved to meet changing requirements and to provide effective international coordination and communication between developers of these standards, stakeholders and the metabolomics community. For example, the Metabolite Identification Task Group will provide engagement with the community on the use of MSI proposed reporting standards for metabolite identification. It is evident that we have reached a time in history where several strands in metabolomics science, including research, application and the emergence of an international network of data exchange through specialist and general purpose data repositories, are converging to bring this field to a new level of professionalism. It is now time to ensure the appropriate development and application of standards in this community.

## Abbreviations

COSMOS: COordination of Standards in MetabOlomicS; InChI: International Chemical Identifier; ISA: Investigation/study/assay; MSI: Metabolomics Standards Initiative; m/z: Mass-to-charge ratio; NIH: National Institutes of Health; SMILES: Simplified Molecular-Input Line-Entry System.

## Competing interests

RMS, CS, MV and WBD have no competing interests. RG is Editor-in-Chief of the journal *Metabolomics*.

## Authors’ contributions

WBD wrote the first draft with input from RMS, CS, MV and RG. All authors have read and approved the final manuscript.
